# An efficient sulfadiazine selection scheme for stable transformation in the model liverwort *Marchantia polymorpha*

**DOI:** 10.1093/jxb/erae256

**Published:** 2024-06-02

**Authors:** Kayla Robinson, Khong-Sam Chia, Alex Guyon, Sebastian Schornack, Philip Carella

**Affiliations:** Cell and Developmental Biology, John Innes Centre, Colney Lane, Norwich, NR4 7UH, UK; Cell and Developmental Biology, John Innes Centre, Colney Lane, Norwich, NR4 7UH, UK; University of Cambridge, Sainsbury Laboratory, Bateman Street, Cambridge, CB2 1LRUK; University of Cambridge, Sainsbury Laboratory, Bateman Street, Cambridge, CB2 1LRUK; Cell and Developmental Biology, John Innes Centre, Colney Lane, Norwich, NR4 7UH, UK; Cardiff University, UK

**Keywords:** Cell biology, evolution, herbicide resistance, *Marchantia polymorpha*, sulfadiazine, transformation

## Abstract

Plant macroevolutionary studies leverage the phylogenetic position of non-flowering model systems like the liverwort *Marchantia polymorpha* to investigate the origin and evolution of key plant processes. To date, most molecular genetic studies in *Marchantia* rely on hygromycin and/or chlorsulfuron herbicide resistance markers for the selection of stable transformants. Here, we used a sulfonamide-resistant *dihydropteroate synthase* (*DHPS*) gene to enable sulfadiazine-based transformation selection in *M. polymorpha*. We demonstrate the reliability of sulfadiazine selection on its own and in combination with existing hygromycin and chlorsulfuron selection schemes through transgene stacking experiments. The utility of this system is further demonstrated through confocal microscopy of a triple transgenic line carrying fluorescent proteins labelling the plasma membrane, cortical microtubules, and the nucleus. Collectively, our findings and resources broaden the capacity to genetically manipulate the increasingly popular model liverwort *M. polymorpha*.

## Teaser

A sulfonamide-resistance gene enables sulfadiazine-based transformation selection in *Marchantia polymorpha* , broadening the capacity to genetically manipulate liverworts.

## Introduction

Bryophytes are a monophyletic clade of non-vascular/non-seed land plants that emerged from algal progenitors over 500 million years ago (Morris *et al.*, 2018; Puttick *et al.*, 2018) and include mosses, liverworts, and hornworts that are sister to all major tracheophytes (vascular plants) (Harris *et al.*, 2022). Given their evolutionary position, bryophytes are often leveraged as evolutionarily insightful platforms to interrogate the molecular genetic principles underpinning plant development, cell biology, physiology, and host–microbe interactions (Bowman *et al.*, 2022; Naramoto *et al.*, 2022; Frangedakis *et al.*, 2023; Jeong *et al.*, 2023).

The model liverwort *Marchantia polymorpha* has emerged as a robust system to explore conserved and derived principles in plant biology (Bowman *et al.*, 2022). Research in this system is facilitated by efficient genetic transformation and CRISPR/Cas-mediated gene editing technologies (Kubota *et al.*, 2013; Tsuboyama and Kodama, 2014; Sugano *et al.*, 2018). To date, the stable genetic transformation of liverworts has generally relied on two key selection schemes based on resistance against the herbicides hygromycin B or chlorsulfuron. Selection using gentamycin or geneticin (G418) resistance is also available (Ishizaki *et al.*, 2015), but this is often less efficient compared with hygromycin or chlorsulfuron (Tsuboyama *et al.*, 2018). As the field continues to grow, there is a concurrent need to expand the range of herbicides that can be used for the stable transformation of multiple transgenes.

Sulfonamides are a class of molecules that suppress plant and bacterial growth by inhibiting the folic acid biosynthesis enzyme dihydropteroate synthase (DHPS) (Brown, 1962; Iwai *et al.*, 1962; Yun *et al.*, 2012). In plants, resistance against sulfonamides like sulfadiazine can be achieved by expressing a sulfonamide-resistant variant of bacterial DHPS (*sul*) that escapes chemical inhibition (Guerineau *et al.*, 1990). Consequently, sulfonamide-resistant *DHPS* has been leveraged to enable the selection of stably transformed Arabidopsis, tomato, and algae on sulfadiazine-containing media (Guerineau *et al.*, 1990; Tabatabaei *et al.*, 2019).

Here, we extend the *sul* DHPS enzyme to *Marchantia* to enable sulfadiazine-mediated selection of stable transgenes. We modified the well-established pMpGWB Gateway binary vector series to incorporate sulfadiazine resistance, which enabled sulfadiazine transformation selection at a level comparable to hygromycin B. We further demonstrate that the pMpGWB series vectors can be stacked into triple transgenic lines carrying resistance against hygromycin, chlorsulfuron, and sulfadiazine. This system provides further flexibility for the genetic manipulation of the model liverwort *M. polymorpha.*

## Materials and methods

### Plant growth

*Marchantia polymorpha* [accessions: Takaragaike-1 (Tak-1), Takaragaike-2 (Tak-2), Cambridge-1 (Cam-1), and Cambridge-2 (Cam-2)] were cultivated axenically from gemmae and grown under a long day photoperiod (16 h light at 60–80 μmol m^–2^ s^–1^ light intensity) on half-strength Murashige and Skoog (MS) medium (pH 6.7) with B5 vitamins at 22 °C.

### Confocal microscopy

Confocal laser scanning microscopy was performed with a Leica Stellaris 8 FALCON equipped with HyDS2 detectors. A white light laser was used to sequentially visualize mCerulean (excited at 440 nm; collected at 455–490 nm), green fluorescent protein (GFP; excited at 488 nm; collected at 505–537 nm) and mScarlet (excited at 561 nm; collected at 574–621 nm). Three independent lines of all newly generated transgenics were analysed, and included at least three biological replicates (*M. polymorpha* gemmae). Microscopy was performed on at least three independent transgenic lines per genotype, imaging at least three gemmae per line. All experiments were performed at least two times with similar results.

### Generating the pMpGWBs00 vector series

To generate the sulfadiazine-resistant pMpGWBs00 vector backbone, we first PCR amplified *Xho*I-flanked *tp-SulR* (oligos in Supplementary Table S1) from an Arabidopsis GABI-KAT (Kleinboelting *et al.*, 2012) mutant (GK-403C01) using Q5 high-fidelity DNA polymerase (NEB M0491) and subsequently performed restriction enzyme digestion and DNA ligation to clone *tp-SulR* into the hygromycin B resistance gene site in pMpGWB100. To generate a Gateway destination vector carrying sulfadiazine resistance, we cloned the 35S promoter–Gateway recombination cassette module from pMpGWB102 into the pMpGWBs00 backbone using unique *Hin*dIII/*Sac*I restriction enzyme sites, following established protocols for swapping Gateway modules into pMpGWB backbones (Ishizaki *et al.*, 2015). All DNA vectors are described in Supplementary Table S2.

### Type IIS and Gateway cloning

The NLS-mCerulean construct was cloned as a Gateway compatible entry clone using type IIS modular cloning (MoClo) by assembling the *att*L1 recombination site (PCR amplified; Supplementary Table S1), mCerulean (pAGM3221), Sv40 NTAG nuclear localization signal (NLS) (pICSL30 043; where NLS is MASSPPKKKRKVSWKM), and *att*L2 recombination site (PCR amplified; Supplementary Table S1) into the pICH47742 acceptor plasmid (Addgene no. 48001) (Weber *et al.*, 2011). A sequence-verified construct was then used for LR recombination (LR Clonase II; Thermo Fisher Scientific) into pMpGWBs02 to generate the 35S::NLS-mCeruluean following the manufacturer’s instructions. Similarly, plasma membrane marker constructs (myrisolated-mScarlet) were generated by LR recombination of an existing pENTR-myr-mScarlet vector into pMpGWB102 (Addgene no. 68556; Ishizaki *et al.*, 2015), pMpGWB303 (Addgene no. 68631; Ishizaki *et al.*, 2015), or pMpGWBs02 (this study). Where appropriate, plasmids were transformed into *Agrobacterium tumefaciens* GV3101 (pMP90) by electroporation for later use in *Marchantia* transformation.

### *Marchantia* transformation

*Marchantia polymorpha* transformation (Tak-1 or the MpEF1a::my-mScarlet/MpEF1a::GFP-MpTUB1 transgenic line; Bonfanti *et al.*, 2023) was performed using the *Agrobacterium*-mediated thallus regeneration protocol (Kubota *et al.*, 2013). Transformants were selected on solid half-strength MS-B5 medium supplemented with cefotaxime (Duchefa C0111: 125 μg ml^–1^) and hygromycin B (Duchefa H0192: 15–25 µg ml^–1^), chlorsulfuron (Duchefa C0177: 0.5–1 μM) and/or sulfadiazine (Sigma-Aldrich S6387: 5 µg ml^–1^) as needed. Stable transgenic liverworts were obtained by propagating gemmae from T_1_ thalli. All experiments were performed in the G_2_ (second asexual/gemma generation) or in subsequent generations. Statistical analysis (Student’s *t*-test, *P*<0.05) of transformation efficiencies was performed in Microsoft Excel.

## Results and discussion

### *Marchantia polymorpha* is sensitive to sulfadiazine

**Fig. 1.**
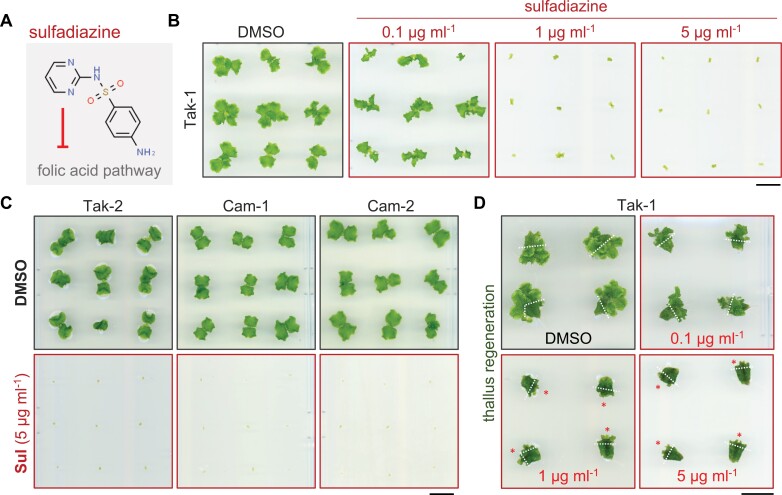
Sulfadiazine impairs *Marchantia* growth and regeneration. (A) Chemical structure of sulfadiazine and a simple schematic illustration indicating its mode of action in inhibiting the folic acid pathway. (B) Dose-dependent sensitivity of wild-type *Marchantia polymorpha* Tak-1 gemmae to increasing concentrations of sulfadiazine relative to a dimethyl sulfoxide (DMSO) negative control (*n*≥9 per condition). Representative images show liverworts 2 weeks post-plating. Performed three times with similar results. Scale bar: 1 cm. (C) Sulfadiazine impairs the growth of male and female genotypes in the Tak and Cam accession backgrounds. Images show Tak-2, Cam-1, and Cam-2 liverworts grown on sulfadiazine (5 µg ml^−1^) or DMSO (*n*=25 per condition). Representative images show liverworts 2 weeks post-plating. Performed three times with similar results. Scale bar: 1 cm. (D) Dose-dependent impairment of *M. polymorpha* Tak-1 thallus regeneration by increasing concentrations of sulfadiazine. Healthy 2.5-week-old Tak-1 thalli were sectioned (dashed lines), transferred to the indicated media, and imaged 2-weeks later (*n*=16 per condition). Asterisks indicate sites of regeneration inhibition. Performed three times with similar results. Scale bar: 1 cm.

To explore whether sulfonamides can function as a selective agent for liverwort transformation, we first tested the sensitivity of *M. polymorpha* gemmae to increasing concentrations of sulfadiazine (Fig. 1A). At high concentrations (1 and 5 µg ml^–1^) sulfadiazine caused severe growth arrest in wild-type (Tak-1) gemmae, whereas a lower concentration (0.1 µg ml^−1^) exerted minimal impact on liverwort growth similar to the dimethyl sulfoxide (DMSO) control (Fig. 1B). Additional *M. polymorpha* accessions (Tak-2, Cam-1, and Cam-2) were similarly impacted by sulfadiazine, as gemmae from all tested backgrounds failed to develop in the presence of sulfadiazine (5 µg ml^–1^) while DMSO controls remained healthy (Fig. 1C). We then assessed whether sulfadiazine restricts the growth of regenerating *M. polymorpha* thalli, which is a pre-requisite for the downstream use of sulfadiazine for stable transformation selection of thalli. Similar to *Marchantia* gemmae, wild-type Tak-1 thallus regeneration was severely impacted at 1 and 5 µg ml^−1^ sulfadiazine, whereas a lower concentration (0.1 µg ml^–1^) partially suppressed regeneration and DMSO had no impact (Fig. 1D). Collectively, these results demonstrate that *Marchantia* is sensitive to the herbicide sulfadiazine.

### Generating a sulfadiazine resistant pMpGWBs00 vector for *Marchantia* transformation

**Fig. 2.**
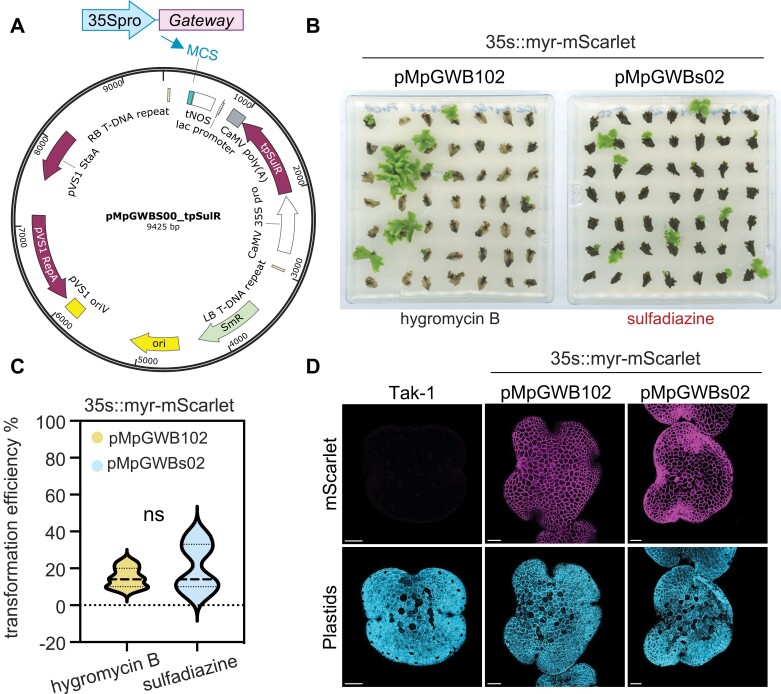
Sulfadiazine-resistant DHPS is suitable for *Marchantia* transformation. (A) Schematic depiction of the construction of a Gateway-compatible vector using the sulfadiazine selection backbone pMpGWBs00. The ‘35Spro::Gateway’ module is cloned into the pMpGWBs00 multiple cloning site (MCS). (B) Stable *Agrobacterium*-mediated transformation of 35S::myr-mScarlet in regenerating *M. polymorpha* Tak-1 liverworts using hygromycin B (pMpGWB102) or sulfadiazine (pMpGWBs02) selection. Images show transformed thalli 6 weeks after selection on herbicide-containing growth media. Performed at least three times with similar results. (C) Transformation efficiency of 35S::myr-mScarlet constructs in the pMpGWB102 (hygromycin B) and pMpGWBs02 (sulfadiazine) vector backbones. The data represent the percentage of validated transformants (strong red fluorescence of thalli) relative to the total number of thalli plated per independent transformation experiments. ns, not statistically different (Student’s *t*-test, *P*<0.05). Data include values from three independent transformation experiments. (D) Confocal microscopy of wild-type (Tak-1) and transgenic 35S::myr-mScarlet liverworts generated using pMpGWB102 or pMpGWBs02 vector. Plastid autofluorescence (cyan) and mScarlet fluorescence (magenta) are displayed. Performed at least twice with similar results. Scale bars: 100 µm.

To investigate whether sulfadiazine is suitable for *Marchantia* transformation selection, we first modified the pMpGWB100 vector backbone by replacing the hygromycin B resistance gene *hpt* with a sulfadiazine resistant version of *DHPS* that is targeted to plastids (*tp-SulR*). The resulting pMpGWBs00 vector was further adapted for use in Gateway cloning by transferring a module containing the strong constitutive 35S promoter and Gateway cloning cassette into a multiple cloning site (MCS) flanked by the nopaline synthase (NOS) terminator (Fig. 2A). To test whether the resulting pMpGWBs02 construct was viable for *Marchantia* transformation, we subcloned a plasma-membrane-targeted myristolated-mScarlet (myr-mScarlet) fluorophore into pMpGWBs02 and transformed it into wild-type Tak-1 using the thallus regeneration method. As a comparison, we also transformed plants with 35S::myr-mScarlet in the pMpGWB102 vector background that carries hygromycin resistance. Over multiple transformation experiments, transgenic lines expressing 35S::myr-mScarlet were obtained through thallus transformations using the pMpGWBs02 (sulfadiazine selection) and the pMpGWB102 (hygromycin B selection) vectors (Fig. 2B). In our conditions, no significant difference in transformation efficiency was observed for hygromycin- or sulfadiazine-based transformation in Tak-1 (Fig. 2C). Moreover, confocal fluorescence microscopy confirmed the stable expression of myr-mScarlet in gemmae of transgenic *Marchantia* generated using pMpGWBs02 or pMpGWB102 vector, whereas untransformed wild-type Tak-1 gemmae did not exhibit mScarlet signals (Fig. 2D). Taken together, our results confirm the viability of the pMpGWBs00 backbone and indicate that sulfadiazine is a suitable selectable marker for *Marchantia* transformation. Future efforts to improve this system could be focused on directing sulfadiazine-resistant DHPS to the mitochondria rather than the chloroplast, as this has been shown to improve transformation efficiency in the angiosperm *Nicotiana tabacum* and the chlorophyte alga *Chlamydomonas reinhardtii* (Tabatabaei *et al.*, 2019).

### Sulfadiazine selection is compatible with hygromycin and chlorsulfuron

**Fig. 3.**
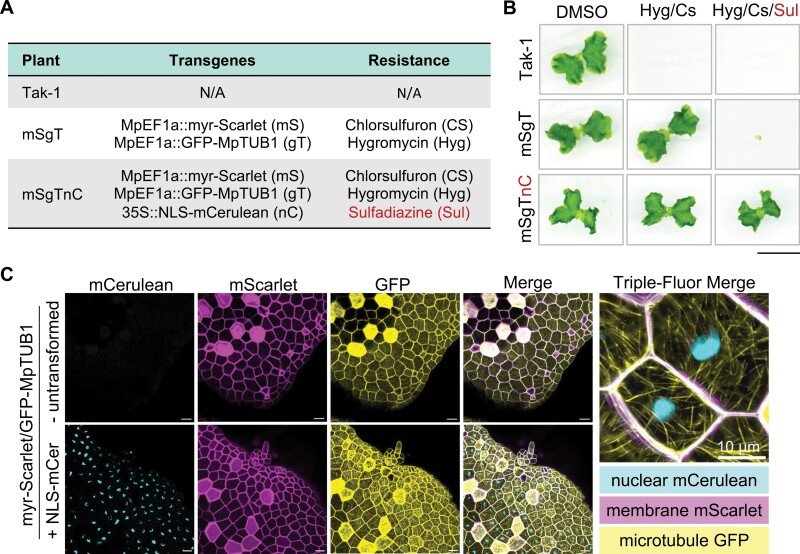
Sulfadiazine-enabled transgene stacking for enhanced cell biology in *Marchantia.* (A) Plant genotypes, their transgenes, and herbicide selection markers. Wild-type Tak-1 serves as an untransformed control, the double transformant mSgT (mS: 35S::myr-mScarlet; gT: MpEF1a::GFP-MpTUB1) carries both hygromycin (Hyg) and chlorsulfuron (CS) selection markers, and the triple transgenic mS-gT-nC carries the 35S::NLS-mCerulean transgene (Sul: sulfadiazine resistance) in the mSgT background. (B) Herbicide resistance selection of *M. polymorpha* wild-type Tak-1, mSgT, and mSgTnC lines on media containing DMSO (control), chlorsulfuron and hygromycin (CS/Hyg), or chlorsulfuron, hygromycin, and sulfadiazine (CS/Hyg/Sul). Images were taken 14 d after gemmae were plated onto the media. Performed twice with similar results. Scale bar: 1 cm. (C) Confocal microscopy showing myr-mScarlet, GFP-MpTUB1, and NLS-mCerulean signals in the mSgTnC triple transgenic relative to the untransformed mSgT background. Scale bars: 20 µm unless shown otherwise.

To test whether sulfadiazine selection is compatible with the commonly used hygromycin B or chlorsulfuron herbicides, we performed transgene stacking experiments using all three selectable markers to stably express three distinct fluorescent proteins targeted to different cellular compartments. To achieve this, we generated a nuclear localization sequence (NLS)-tagged mCerulean marker and cloned it into pMpGWBs02. The resulting 35S::NLS-mCerulean (nC) construct carrying sulfadiazine resistance was then transformed into an existing transgenic liverwort carrying the plasma membrane marker MpEF1a::myr-mScarlet (mS; chlorsulfuron resistance) and MpEF1a::GFP-MpTUB1 (gT; hygromycin B resistance) (Buschmann *et al.*, 2016; Bonfanti *et al.*, 2023). We successfully recovered transgenics carrying all three transgenes (referred to as mSgTnC) and compared their sensitivity to triple selection in comparison with an untransformed wild-type Tak-1 control and the untransformed mSgT double transgenic background (Fig. 3A). Wild-type Tak-1 grew normally on DMSO control medium but failed to grow on media containing combinations of hygromycin and chlorsulfuron (Hyg/Cs) or all three herbicides (Hyg/Cs/Sul) (Fig. 3B). In comparison, mSgT grew on media supplemented with Hyg/Cs or DMSO but was sensitive to the addition of sulfadiazine. As expected, the stacked transgenic line mSgTnC grew in all conditions as it carries resistance to all three herbicides. While mSgTnC liverworts grew to a typical size on triple selection, qualitative aspects of liverwort development appeared slightly altered, as the area around the apical notch was less rounded than in plants grown on DMSO control medium. We advise caution in performing experiments on liverworts directly grown on medium with multiple herbicides.

### Cell biology using stacked fluorescent markers

Stacked transgenics enabled high resolution confocal microscopy experiments to visualize three distinct subcellular features in liverwort cells. In the mSgTnC line, laser scanning confocal fluorescence microscopy demonstrated membrane-localized mScarlet accumulation, GFP-labelled microtubules, and nuclear localized mCerulean signals (Fig. 3C). Signals for mCerulean were specific to the presence of the NLS-mCerulean construct, as the untransformed mSgT line only exhibited mScarlet and GFP signals and not mCer (Fig. 3C). High resolution imaging of mSgTnC gemmae clearly labelled nuclei, the plasma membrane, and cortical microtubule filaments (Fig. 3C). These results demonstrate that pMpGWBs00 vectors can be used to stack multiple transgenes for multi-fluorescence cell biology experiments in *Marchantia.*

### Conclusions

In this study, we demonstrate that the model liverwort *M. polymorpha* is susceptible to the sulfonamide herbicide sulfadiazine, which enabled transformation selection through the transfer of the sulfadiazine-resistant DHPS resistance marker into the well-established pMpGWB background (Ishizaki *et al.*, 2015). Sulfadiazine-based selection through pMpGWBs00 vectors further extends the range of transformation selection schemes in liverworts, which is beneficial since selection using gentamycin or geneticin (G418) is less efficient than that with the commonly used herbicides hygromycin B and chlorsulfuron (Tsuboyama *et al.*, 2018). We expect this to be particularly useful for transgene stacking, which is essential for assaying multiple promoter reporter constructs (promoter::GUS or promoter::fluorophore), localizing the subcellular distribution of multiple proteins, or when rescuing CRISPR/Cas mutants in accessions or genotypes where backcrossing is not possible. In this study, we demonstrated the utility of this system for the subcellular localization of multiple compartments and validated the compatibility of multi-fluorescence microscopy using the fluorophores mCerulean, GFP, and mScarlet. The compatibility of the pMpGWBs00 backbone with existing pMpGWB and pGWB vectors allows for the future transfer of alternative Gateway cloning modules.

## Supplementary data

The following supplementary data are available at *JXB* online.

Table S1. Oligonucleotide primers used in this study.

Table S2. Vectors used in this study.

erae256_suppl_Supplementary_Tables_S1-S2

## Data Availability

All data supporting the conclusions of this study are contained within the manuscript and in the online supplementary data. A detailed protocol for *Marchantia* thallus transformation and sulfadiazine selection is available at Protocols.io (doi: dx.doi.org/10.17504/protocols.io.kqdg329bqv25/v1).

## Abbreviations:

DHPS: dihydropteroate synthase
myr: myristoylation
NLS: nuclear localization sequence.
